# Physical Activity Reduces the Effect of High Body Mass Index on Kidney Stones in Diabetes Participants From the 2007–2018 NHANES Cycles: A Cross-Sectional Study

**DOI:** 10.3389/fpubh.2022.936552

**Published:** 2022-07-01

**Authors:** Weipu Mao, Lei Zhang, Si Sun, Jianping Wu, Xiangyu Zou, Guangyuan Zhang, Ming Chen

**Affiliations:** ^1^People's Hospital of Putuo District, Shanghai, China; ^2^Affiliated Zhongda Hospital of Southeast University, Nanjing, China; ^3^School of Basic Medical Sciences, Weifang Medical University, Weifang, China

**Keywords:** kidney stones, body mass index, physical activity, diabetes, NHANES database

## Abstract

**Background:**

Body mass index (BMI) is a vital risk factor for kidney stones, but physical activity may reduce the incidence of kidney stones. However, it remains unknown whether physical activity reduces the effect of high BMI on kidney stones in diabetes participants.

**Methods:**

We included clinical information from 4,008 adult participants with diabetes from the National Health and Nutrition Examination Survey (NHANES) database from 2007 to 2018. Univariate and multivariate logistic regression analyses were used to analyze the relationship between BMI and kidney stones, as well as the risk of BMI and kidney stones in different physical activity subgroups.

**Results:**

A total of 4,008 diabetic participants were included in this study, of whom 652 (16.3%) self-reported a history of kidney stones. Logistic regression analysis showed a positive association between BMI and kidney stones. After adjusting for other confounders, the adjusted ORs for the risk of kidney stones was 1.514 (95% CI, 1.134–2.022, *p* = 0.005) for participants with BMI ≥30 kg/m^2^ among all participants; the risk of kidney stones was elevated (OR = 1.572, 95% CI, 1.134–2.022, *p* = 0.005) in group without physical activity, and a reduced risk (OR = 1.421, 95% CI, 0.847–2.382, *p* = 0.183) in the group with physical activity. Furthermore, similar results were found in most subgroups.

**Conclusion:**

Our study suggests that high BMI is a risk factor for diabetes kidney stone participants and that physical activity may moderate this relationship to some extent.

## Background

Kidney stones are stones in the junction of renal calyces, renal pelvis or pelvic ureter and are one of the most common urological diseases, accounting for 40–50% of all urinary stone diseases (1, 2). Hematuria and back pain are the main clinical manifestations, which can cause urological infection, urinary tract obstruction and even uremia (3, 4). Kidney stones are a complex disease caused by environmental, dietary and genetic factors, with an incidence of over 6–12% and a 5-year recurrence rate of up to 50%, seriously affecting human health (5).

There are many risk factors for kidney stones, such as metabolic, dietary, pharmacological and environmental (6, 7). Metabolic syndrome is a group of clinical symptoms of metabolic disorders characterized by abdominal obesity, hyperglycemia, hypertension, high triglycerides, and abnormal HDL cholesterol (8). Related studies have found that the development of kidney stones is closely related to lifestyle-related diseases such as hyperglycemia, obesity, hypertension and dyslipidemia components of the metabolic syndrome, and that the metabolic syndrome and its components can significantly increase the prevalence of kidney stones (9, 10).

Diabetes is a common metabolic disease, and previous studies have shown a positive association between diabetes and kidney stone risk (11). Taylor et al. (12) found that diabetic patients are more likely to develop kidney stones than the general population. Domingos et al. (13) found a higher prevalence of diabetes in patients with kidney stones compared to normal subjects through a survey analysis of 23,349 individuals. Patients with diabetic kidney stones have a worse prognosis and poorer quality of life than patients with general kidney stones (14).

Obesity is a common disease worldwide and is often defined by body mass index (BMI). One study found a correlation between the incidence of kidney stones and human BMI, with both overweight (BMI 25.0–29.9 kg/m^2^) and obesity (BMI ≥30 kg/m^2^) increasing the risk of kidney stones to some extent (15). Obesity is not only associated with the formation of kidney stones but also with their recurrence. Lee et al. (16) analyzed more than 700 patients with primary kidney stones and found that the proportion of stone recurrence within 5 years was significantly higher in patients with BMI ≥30 kg/m2 than in those with BMI <25 kg/m2 (42 vs. 14.9%, *p* = 0.0012), and multiple logistic regression analysis showed obesity was the only predictor of stone recurrence.

A healthy lifestyle can improve quality of life and reduce the incidence of disease, and physical activity is an integral aspect of this (17). However, studies on the relationship between physical activity and kidney stones are relatively few and the results are inconsistent. Feng et al. (18) found that physical activity was inversely associated with the prevalence of kidney stones through a survey of 8,931 US participants, and that physical activity reduced the incidence of kidney stones. In contrast, in a large prospective cohort, no independent association between physical activity and kidney stones was found (19). The specific relationship between physical activity levels and kidney stones is not known.

Since most of the current study population is general kidney stone patients, fewer studies have been conducted on diabetes kidney stone patients. In addition, since both BMI and physical activity may have an effect on kidney stones, it is unclear whether they have a combined effect in modulating diabetes kidney stones. In this study, we used data from the National Health and Nutrition Examination Survey (NHANES) database to explore the relationship between BMI and patients with diabetes kidney stones and to provide insight into whether physical activity may reduce the risk of kidney stones caused by high BMI.

## Materials and Methods

### Data Sources and Preparation

The NHANES database is a cross-sectional survey conducted by the National Center for Health Statistics (NCHS) to assess the health and nutritional status of adults and children in the U.S. The NHANES database includes demographic information, socioeconomic information, dietary status, health-related issues, and medical health-related physical examinations and laboratory tests (20). The NHANES database is a publicly available database that the database has published data files from the survey online on a 2-year cycle since 1999.

**Figure 1:**
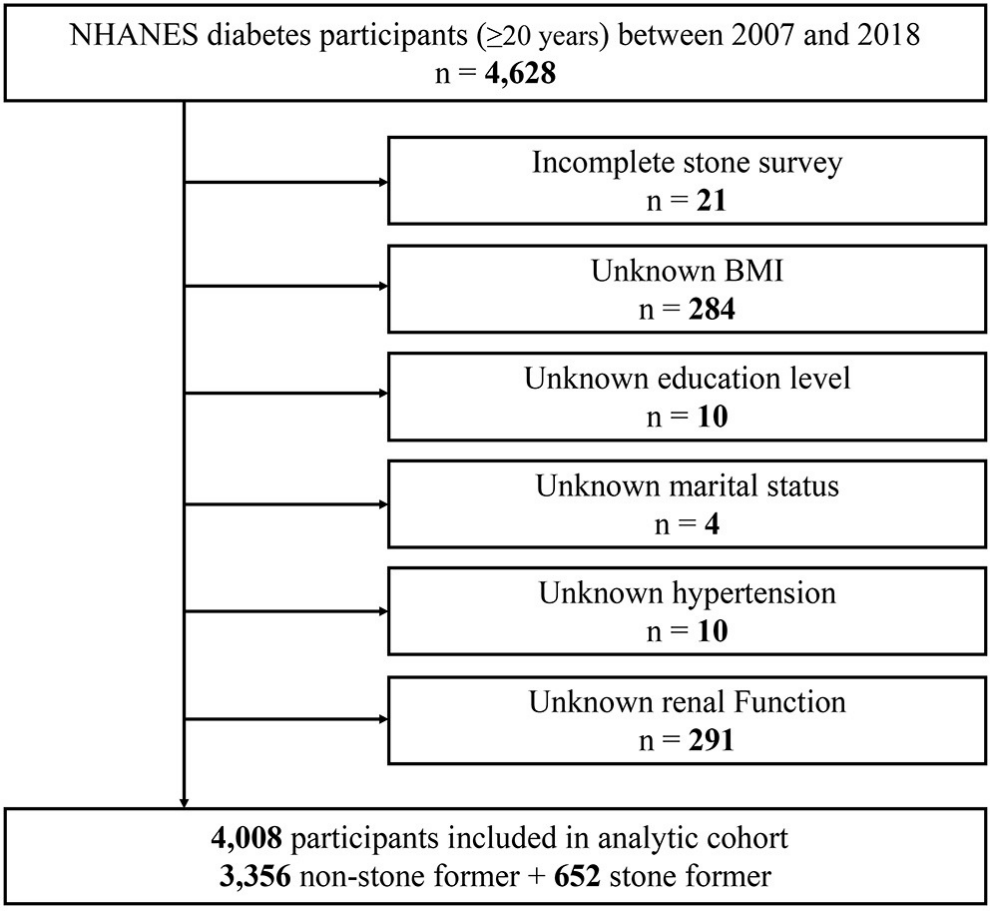
Schematic overview for patient identification.

The current study included publicly available data from NHANES for six cycles between 2007 and 2018. We first identified 4,628 adult participants with diabetes and subsequently developed the following exclusion criteria: a) incomplete stone survey (*n* = 21); b) unknown BMI (*n* = 284); c) unknown education level (*n* = 10); d) unknown marital status (*n* = 4); e) unknown hypertension status (*n* = 10); f) unknown renal function indicators (*n* = 291). A total of 4,008 study subjects were eventually enrolled in this study (Figure 1).

### Study Variables and Other Variables

Kidney stones were determined based on the KIQ026 question from the Kidney Conditions - Urology survey in the questionnaire data. Questions on kidney stones was asked in the home, by trained interviewers, using the Computer-Assisted Personal Interview (CAPI) system (21). In the questionnaire, participants were asked by a trained professional, “Have you ever had a kidney stone” and if the participant answered “Yes,” they were considered to have a history of kidney stones.

In addition, some other variables such as gender (female and male), age (<60 years and ≥60 years), race (Non-Hispanic white, Non-Hispanic black, Mexican American, other Hispanic, and other race), education level (less than high school, high school or equivalent, and college or above), marital status (married and unmarried), BMI (<25.0 kg/m^2^ 25.0–29.9 kg/m^2^ and ≥30.0 kg/m^2^), hypertension (yes and no), smoking status (never, former and current), physical activities status (yes and no), and blood urea nitrogen, creatinine, uric acid, and estimated glomerular filtration rate (eGFR) were also included in this study. BMI was calculated by the formula: weight(kg)/(height(m^2^)^*^height(m^2^)). Physical activity was determined according to “Physical Activity” in the questionnaire data. Hypertension and diabetes were diagnosed by a physician or other health professional. The eGFR was calculated by the following Equation (4):


$$\begin{array}{l} eGFR=141\times min{\left(Scr/\alpha ,1\right)}^{\beta }\times max{\left(Scr/\alpha ,1\right)}^{-1.209}\times 0.993^{\text{age}}\\ \;\times \gamma \times 1.159(ifblack).\\ Male:\;\alpha =0.9,\beta =-0.411,\alpha =1,\\ Female:\alpha =0.7,\beta =-0.329,\alpha =1.018. \end{array}$$

### Statistical Analysis

Continuous data were presented as mean ± standard deviation (SD) and categorical data were described as number (n) and percentage (%). *T*-tests were used to assess continuous variables and chi-square tests were used to assess categorical demographic differences. In processing the data, weights were analyzed for the data in different cycles. Logistic regression models and dose-response curves with restricted cubic spline (RCS) were used to assess the association between BMI and kidney stones in different physical activity groups, and results are presented as adjusted odds ratios (aORs) and 95% confidence intervals (CIs). We constructed four models for the logistic regression analysis. Model 1 was univariate analysis. In model 2, we adjusted for gender, age, and race because demographic factors were associated with physical activity. Subsequently, we further adjusted for variables related to participants' living conditions, such as education, marital status, hypertension, and smoking status in model 3. Finally, we included renal function-related indicators (blood urea nitrogen, creatinine, uric acid and eGFR) in model 4. R software (version 3.5.3) and SPSS software (version 24.0) were applied in the present study, and *P*-values calculated at <0.05 were considered statistically significant.

## Results

Table 1 demonstrates the clinicopathological characteristics of all participants. A total of 4,008 diabetes participants were enrolled in the study between 2007 and 2018, of whom 652 (16.3%) self-reported a history of kidney stones and 3,356 (83.7%) had no history of kidney stones. Chi-square test revealed significant differences between stone formers and non-stone formers groups on the variables of gender, age, race, marital status and BMI. The proportion of stone formers who were male, ≥60 years, Non-Hispanic white, married, and BMI ≥30.0 kg/m^2^ were higher in stone formers than in non-stone formers groups. Stone formers group had higher blood urea nitrogen and creatinine levels and lower eGFR compared to non-stone formers. In addition, among the total participants, 2,671 (66.6%) did not engage in physical activity and 1,337 (33.4%) did not engage in physical activity, with a slightly higher proportion of no physical activity in the stone formers group (*p* = 0.160).

**Table 1:** Baseline characteristics of diabetes participants between 2007 and 2018.

**Characteristic**	**Total**	**None-stone formers**	**Stone formers**	**P-value**
	**No. (%)**	**No. (%)**	**No. (%)**	
Total patients	4,008	3,356 (83.7)	652 (16.3)	
**Gender**				<0.001
Male	2,081 (51.9)	1,684 (50.2)	397 (60.9)	
Female	1,927 (48.1)	1,672 (49.8)	255 (39.1)	
**Age**				0.027
<60 years	1,487 (37.1)	1,270 (37.8)	217 (33.3)	
≥60 years	2,521 (62.9)	2,086 (62.2)	435 (66.7)	
**Race**				<0.001
Non-Hispanic white	1,382 (34.5)	1,072 (31.9)	310 (47.5)	
Non-Hispanic black	1,021 (25.5)	924 (27.5)	97 (14.9)	
Mexican American	711 (17.7)	607 (18.1)	104 (16.0)	
Other Hispanic	433 (10.8)	361 (10.8)	72 (11.0)	
Other	461 (11.5)	392 (11.7)	69 (10.6)	
**Education level**				0.448
Less than high school	1,393 (34.8)	1,179 (35.1)	214 (32.8)	
High school or equivalent	890 (22.2)	746 (22.2)	144 (22.1)	
College or above	1,725 (43.0)	1,431 (42.6)	294 (45.1)	
**Marital status**				0.007
Married	2223 (55.5)	1830 (54.5)	393 (60.3)	
Unmarried	1785 (44.5)	1526 (45.5)	259 (39.7)	
**BMI (kg/m** ^**2**^ **)**				0.017
<25.0	536 (13.4)	464 (13.8)	72 (11.0)	
25.0–29.9	1,154 (28.8)	983 (29.3)	171 (26.2)	
≥30.0	2,318 (57.8)	1,909 (56.9)	409 (62.7)	
**Hypertension**				0.544
Yes	2800 (69.9)	2338 (69.7)	462 (70.9)	
No	1208 (30.1)	1018 (30.3)	190 (29.1)	
**Smoking status**				0.099
Never	2,004 (50.0)	1,699 (50.6)	305 (46.8)	
Former	1,380 (34.4)	1,132 (33.7)	248 (38.0)	
Current	624 (15.6)	525 (15.6)	99 (15.2)	
**Physical activities**				0.160
No	2,671 (66.6)	2,221 (66.2)	450 (69.0)	
Yes	1,337 (33.4)	1,135 (33.8)	202 (31.0)	
Blood urea nitrogen (mg/dL)	17.13, 8.82	16.93, 8.60	18.17, 9.78	0.001
Creatinine (mg/dL)	1.05, 0.77	1.03, 0.73	1.11, 0.98	0.024
Uric acid (mg/dL)	5.74, 1.60	5.73, 1.58	5.80, 1.68	0.363
eGFR [mL/(min·1.73 m^2^)]	80.64, 26.26	81.21, 26.39	77.69, 25.40	0.002

*For categorical variables, P-values were analyzed by chi-square tests. For continuous variables, the t-test was used.*

*BMI, body mass index; eGFR, estimated glomerular filtration rate*.

We also studied the clinicopathological characteristics of the total population according to physical activity (Table 2). The results showed significant differences between the physical activity and no physical activity groups in the variables of gender, age, race, education, BMI, hypertension, smoking status, blood urea nitrogen, creatinine and eGFR. The proportion of <60 years, college or above, BMI <30 kg/m^2^, no hypertension, and never smoking was significantly higher in the physical activity group than in the no physical activity group. In addition, participants in the physical activity group had lower levels of blood urea nitrogen, creatinine and higher levels of eGFR compared to the no physical activity group.

**Table 2:** Baseline characteristics of diabetes participants between 2007 and 2018 based on physical activity status.

**Characteristic**	**Total**	**No physical activity**	**Physical activity**	**P-value**
	**No. (%)**	**No. (%)**	**No. (%)**	
Total patients	4,008	2,671 (66.6)	1,337 (33.4)	
**Gender**				<0.001
Male	2,081 (51.9)	1,311 (49.1)	770 (57.6)	
Female	1,927 (48.1)	1,360 (50.9)	567 (42.4)	
**Age**				<0.001
<60 years	1,487 (37.1)	904 (33.8)	583 (43.6)	
≥60 years	2,521 (62.9)	1,767 (66.2)	754 (56.4)	
**Race**				<0.001
Non-Hispanic white	1,382 (34.5)	951 (35.6)	431 (32.2)	
Non-Hispanic black	1,021 (25.5)	651 (24.4)	370 (27.7)	
Mexican American	711 (17.7)	495 (18.5)	216 (16.2)	
Other Hispanic	433 (10.8)	309 (11.6)	124 (9.3)	
Other	461 (11.5)	265 (9.9)	196 (14.7)	
**Education level**				<0.001
Less than high school	1,393 (34.8)	1,099 (41.1)	294 (22.0)	
High school or equivalent	890 (22.2)	616 (23.1)	274 (20.5)	
College or above	1,725 (43.0)	956 (35.8)	769 (57.5)	
**Marital status**				0.214
Married	2,223 (55.5)	1,463 (54.8)	760 (56.8)	
Unmarried	1,785 (44.5)	1,208 (45.2)	577 (43.2)	
**BMI (kg/m** ^**2**^ **)**				0.010
<25.0	536 (13.4)	347 (13.0)	189 (14.1)	
25.0-29.9	1,154 (28.8)	735 (27.5)	419 (31.3)	
≥30.0	2,318 (57.8)	1,589 (59.5)	729 (54.5)	
**Hypertension**				<0.001
Yes	2,800 (69.9)	1,926 (72.1)	874 (65.4)	
No	1208 (30.1)	745 (27.9)	463 (34.6)	
**Smoking status**				<0.001
Never	2,004 (50.0)	1,284 (48.1)	720 (53.9)	
Former	1,380 (34.4)	928 (34.7)	452 (33.8)	
Current	624 (15.6)	459 (17.2)	165 (15.6)	
Blood urea nitrogen (mg/dL)	17.13, 8.82	17.59, 9.46	16.22, 7.29	<0.001
Creatinine (mg/dL)	1.05, 0.77	1.07, 0.80	1.00, 0.72	0.010
Uric acid (mg/dL)	5.74, 1.60	5.76, 1.65	5.70, 1.50	0.260
eGFR [mL/(min·1.73 m^2^)]	80.64, 26.26	78.52, 26.74	84.86, 24.75	<0.001

*For categorical variables, P-values were analyzed by chi-square tests. For continuous variables, the t-test was used.*

*BMI, body mass index; eGFR, estimated glomerular filtration rate*.

Participants were divided into three groups according to BMI criteria: BMI <25 kg/m^2^, BMI 25.0–29.9 kg/m^2^ and BMI ≥30 kg/m^2^ groups. Among all diabetes participants, univariate logistic regression analysis showed that the risk of kidney stones was 12.1% higher (95% CI, 0.833–1.508, *p* = 0.490) in the BMI 25.0–29.9 kg/m^2^ group and 38.1% higher (95% CI, 1.054–1.809, *p* = 0.019) in the BMI ≥30 kg/m^2^ group compared with the BMI <25 kg/m^2^ group. After adjusting for all other variables, BMI ≥30 kg/m^2^ remained an independent risk factor for kidney stones, with a risk of 1.514 (95% CI, 1.134–2.022, *p* = 0.005) in the BMI ≥30 kg/m2 group compared to the BMI <25 kg/m2 group (Table 3).

**Table 3:** Logistic regression analyzed the relationship between BMI and the presence of kidney stone in different physical activity groups.

**BMI**	**Model 1**		**Model 2**		**Model 3**		**Model 4**	
	**aOR (95% CI)**	***P***	**aOR (95% CI)**	***P***	**aOR (95% CI)**	***P***	**aOR (95% CI)**	***P***
**All participants**		0.017		0.001		0.003		0.001
<25.0 kg/m^2^	Reference		Reference		Reference		Reference	
25.0–30.0 kg/m^2^	1.121 (0.833–1.508)	0.490	1.090 (0.806–1.474)	0.577	1.081 (0.798–1.464)	0.614	1.111 (0.820–1.507)	0.497
≥30.0 kg/m^2^	1.381 (1.054–1.809)	0.019	1.483 (1.119–1.963)	0.006	1.457 (1.096–1.938)	0.010	1.514 (1.134–2.022)	0.005
**No physical activity**		0.015		0.004		0.009		0.004
<25.0 kg/m^2^	Reference		Reference		Reference		Reference	
25.0–30.0 kg/m^2^	1.099 (0.760-1.590)	0.615	1.078–(0.741–1.568)	0.696	1.072 (0.735–1.562)	0.718	1.104 (0.756–1.612)	0.608
≥30.0 kg/m^2^	1.455 (1.044–2.029)	0.027	1.531 (1.087–2.157)	0.015	1.491 (1.053–2.111)	0.025	1.572 (1.103–2.240)	0.012
**Physical activity**		0.711		0.315		0.372		0.342
<25.0 kg/m^2^	Reference		Reference		Reference		Reference	
25.0–30.0 kg/m^2^	1.161 (0.705–1.912)	0.558	1.116 (0.669–1.860)	0.675	1.112 (0.663–1.864)	0.688	1.176 (0.697–1.983)	0.544
≥30.0 kg/m^2^	1.216 (0.763–1.938)	0.411	1.379 (0.841–2.259)	0.202	1.361 (0.819–2.261)	0.234	1.421 (0.847–2.382)	0.183

*Adjusted covariates: model 1: univariate analysis; model 2: gender, age and race; model 3: model 1 plus education level, marital status, hypertension and smoking status; model 4: model 3 plus blood urea nitrogen, creatinine, uric acid and eGFR.*

*PA, physical activities; BMI, body mass index; eGFR, estimated glomerular filtration rate; CI, confidence interval; aOR, adjusted odds ratio*.

**Figure 2:**
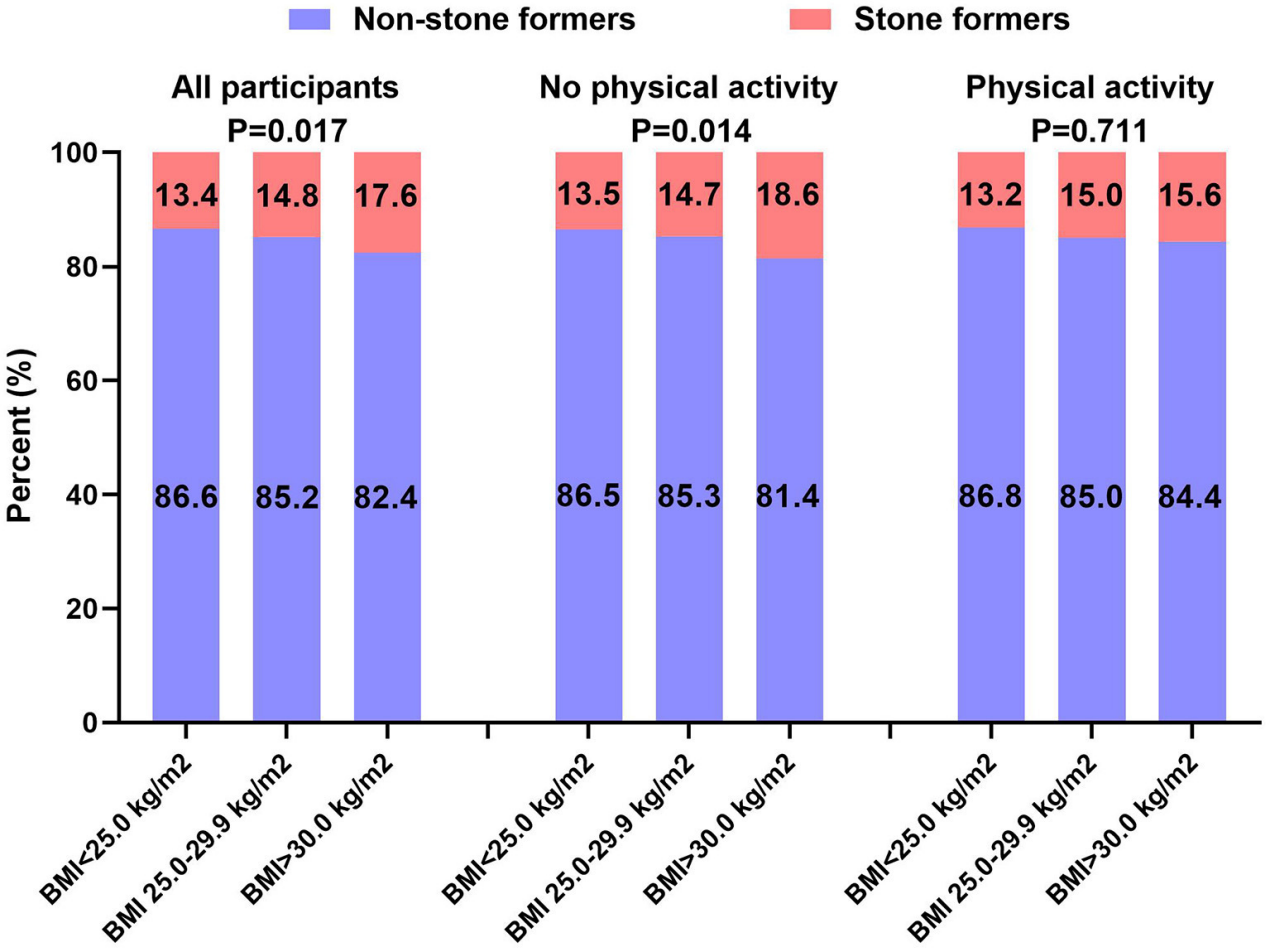
Prevalence of kidney stones in different BMI groups among different physical activity groups.

**Figure 3:**
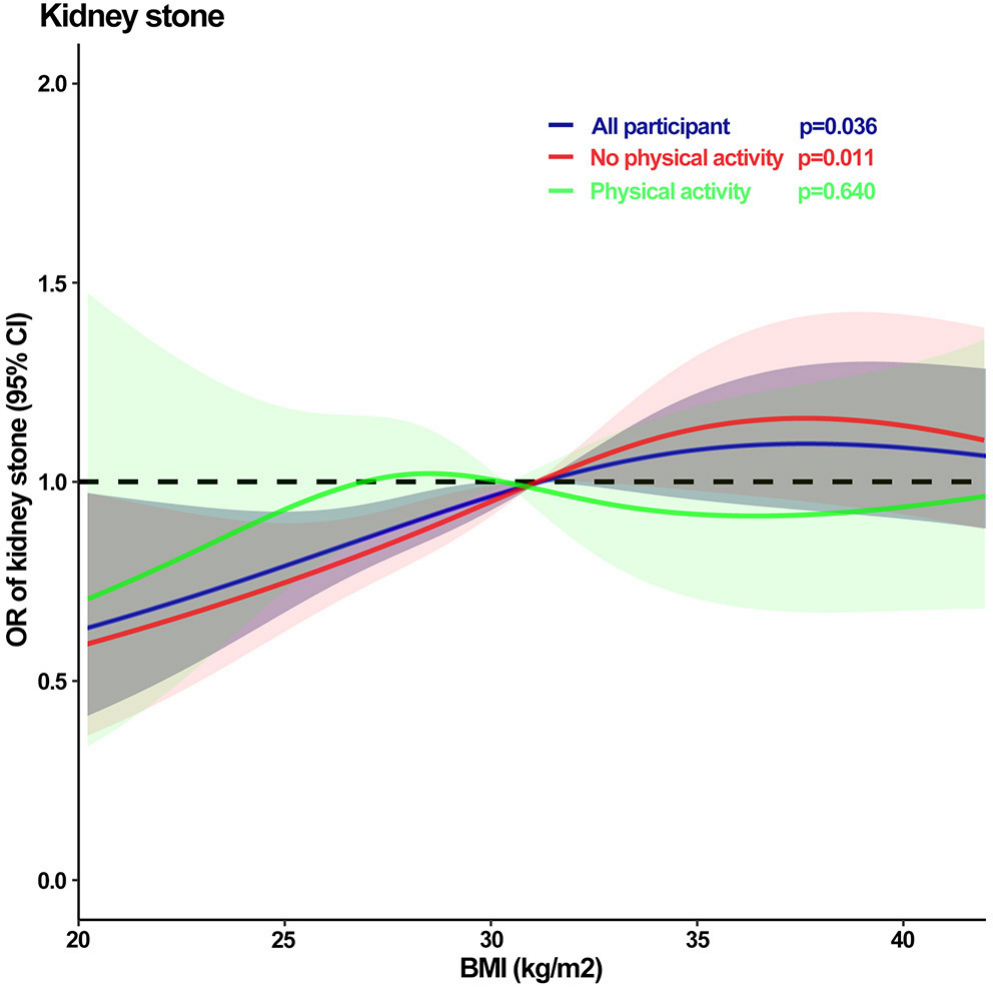
The dose–response analysis of kidney stones with BMI by physical activity type. OR, Odds ratio; CI, Confidence interval.

In addition, we evaluated the effect of physical activity on the relationship between BMI and kidney stones. We found a positive association between BMI and kidney stones in all participants (*p* = 0.017), with a prevalence of kidney stones in the three BMI groups being 13.4, 14.8, and 17.6%, respectively, and the highest prevalence of kidney stones in the BMI ≥30 kg/m^2^ group (Figure 2). However, in the no physical activity group, the prevalence of kidney stones increased to 18.6% in the BMI ≥30 kg/m^2^ group (*p* = 0.017) and decreased to 15.6% in the BMI ≥30 kg/m^2^ group with physical activity (*p* = 0.711). Moreover, dose-response curves showed a correlation between BMI and kidney stones in all participants (*p* = 0.036) and no physical activity group (*p* = 0.011), while no correlation in the physical activity group (*p* = 0.640) (Figure 3).

Table 3 shows the relationship between BMI and kidney stones in different physical activity groups. We found that BMI was an independent risk factor for kidney stones in the no physical activity group, with a risk of 1.572 (95% CI, 1.103–2.240, *p* = 0.012) for kidney stones in the BMI ≥30 kg/m^2^ group compared to the BMI <25 kg/m^2^ group. In contrast, in the physical activity group, BMI was not an independent risk factor for kidney stones (*p* = 0.342), and the risk of kidney stones in the BMI ≥30 kg/m^2^ group was 1.421 compared to the BMI <25 kg/m^2^ group (*p* = 0.183, no statistically significant). In addition, the risk of kidney stones in the BMI ≥30 kg/m2 group was also found to be lower in the physical activity group than in the no physical activity group in most subgroup analyses (Table 4).

**Table 4:** Subgroup analyses between BMI and the presence of kidney stone in NHANES 2007–2018.

**Subgroups**	**BMI (kg/m** ^**2**^ **) [aOR (95% CI)]**			***P***
	** <25.0**	**25.0–29.9**	**≥30.0**	
**Gender**				
Male				
All patients	1.000	1.216 (0.834–1.774)	1.493 (1.030–2.163)	0.068
No physical activity	1.000	1.271 (0.779–2.073)	1.661 (1.032–2.673)	0.066
Physical activity	1.000	1.258 (0.681–2.323)	1.383 (0.743–2.574)	0.591
Female				
All patients	1.000	0.919 (0.540–1.561)	1.477 (0.922–2.365)	0.019
No physical activity	1.000	0.889 (0.481–1.643)	1.490 (0.867–2.562)	0.034
Physical activity	1.000	0.996 (0.336–2.951)	1.472 (0.542–3.998)	0.504
**Age**				
<60 years				
All patients	1.000	0.921 (0.513–1.652)	1.434 (0.847–2.427)	0.060
No physical activity	1.000	1.147 (0.434–3.033)	1.971 (0.792–4.901)	0.143
Physical activity	1.000	0.827 (0.392–1.748)	1.209 (0.624–2.342)	0.342
≥60 years				
All patients	1.000	1.192 (0.831–1.708)	1.517 (1.069–2.151)	0.028
No physical activity	1.000	1.213 (0.779–1.889)	1.692 (1.109–2.581)	0.014
Physical activity	1.000	1.248 (0.655–2.378)	1.241 (0.643–2.399)	0.780
**Race**				
Non-Hispanic White				
All patients	1.000	0.775 (0.484–1.240)	1.166 (0.757–1.796)	0.044
No physical activity	1.000	0.736 (0.416–1.302)	1.221 (0.727–2.050)	0.045
Physical activity	1.000	0.906 (0.376–2.181)	1.213 (0.527–2.793)	0.593
Non-Hispanic Black				
All patients	1.000	1.368 (0.544–3.440)	2.251 (0.980–5.170)	0.059
No physical activity	1.000	3.572 (0.763–16.728)	6.401 (1.492–27.454)	0.017
Physical activity	1.000	0.649 (0.163–2.573)	0.880 (0.257–3.011)	0.789
Others				
All patients	1.000	1.435 (0.908–2.266)	1.633 (1.034–2.577)	0.109
No physical activity	1.000	1.817 (0.816–4.044)	1.840 (0.802–4.221)	0.303
Physical activity	1.000	1.276 (0.722–2.257)	1.414 (0.808–2.474)	0.474
**Education**				
Less than high school				
All patients	1.000	1.061 (0.637–1.767)	1.572 (0.975–2.535)	0.041
No physical activity	1.000	1.106 (0.621–1.969)	1.647 (0.965–2.813)	0.056
Physical activity	1.000	0.771 (0.244–2.430)	1.127 (0.360–3.524)	0.706
High school or equivalent				
All patients	1.000	0.908 (0.492–1.677)	1.092 (0.607–1.967)	0.695
No physical activity	1.000	0.725 (0.337–1.560)	1.090 (0.532–2.234)	0.320
Physical activity	1.000	1.186 (0.396–3.550)	0.906 (0.299–2.746)	0.782
College or above				
All patients	1.000	1.297 (0.792–2.124)	1.728 (1.078–2.769)	0.036
No physical activity	1.000	1.496 (0.747–2.995)	1.976 (1.022–3.819)	0.093
Physical activity	1.000	1.346 (0.642–2.820)	1.934 (0.939–3.983)	0.132
**Marital status**				
Married				
All patients	1.000	1.095 (0.736–1.630)	1.446 (0.980–2.134)	0.052
No physical activity	1.000	1.234 (0.737–2.066)	1.683 (1.021–2.773)	0.051
Physical activity	1.000	0.960 (0.506–1.821)	1.171 (0.615–2.232)	0.686
Unmarried				
All patients	1.000	1.139 (0.703–1.846)	1.652 (1.062–2.571)	0.020
No physical activity	1.000	1.863 (0.702–4.944)	2.224 (0.858–5.763)	0.258
Physical activity	1.000	0.979 (0.554–1.730)	1.508 (0.906–2.513)	0.058
**Hypertension**				
Yes				
All patients	1.000	0.951 (0.648–1.394)	1.308 (0.917–1.865)	0.028
No physical activity	1.000	1.029 (0.515–2.057)	1.315 (0.681–2.537)	0.493
Physical activity	1.000	0.916 (0.575–1.460)	1.282 (0.834–1.972)	0.073
No				
All patients	1.000	1.395 (0.838–2.322)	1.900 (1.147–3.146)	0.032
No physical activity	1.000	1.561 (0.807–3.022)	2.370 (1.254–4.478)	0.019
Physical activity	1.000	1.202 (0.527–2.743)	1.404 (0.590–3.343)	0.735
**Smoking status**				
Never				
All patients	1.000	1.137 (0.717–1.804)	1.709 (1.102–2.651)	0.007
No physical activity	1.000	1.208 (0.674–2.164)	1.728 (0.997–2.996)	0.056
Physical activity	1.000	1.117 (0.516–2.417)	1.804 (0.853–3.814)	0.112
Former				
All patients	1.000	1.023 (0.620–1.689)	1.244 (0.771–2.006)	0.432
No physical activity	1.000	0.922 (0.503–1.692)	1.241 (0.704–2.186)	0.329
Physical activity	1.000	1.469 (0.564–3.827)	1.247 (0.474–3.282)	0.708
Current				
All patients	1.000	1.148 (0.555–2.376)	1.540 (0.776–3.055)	0.363
No physical activity	1.000	1.262 (0.491–3.241)	2.120 (0.88–5.057)	0.132
Physical activity	1.000	0.725 (0.208–2.531)	0.704 (0.193–2.573)	0.850

*Adjusted covariates: gender, age, race, education levels, marital status, hypertension, smoking status, blood urea nitrogen, creatinine, uric acid, and estimated glomerular filtration rate (eGFR). aOR, adjusted odds ratio; CI, confidence interval*.

## Discussion

In the current study, we explored the relationship between physical activity, BMI and kidney stones using clinical data from the NHANES database of participants with diabetes kidney stones during 2007–2018. We first investigated the relationship between BMI and kidney stones: multivariate logistic regression revealed that the risk of kidney stones increased with increasing BMI and that BMI was an independent risk factor for kidney stones. Subsequently, we explored the effect of physical activity and found that physical activity reduced the effect of high BMI on kidney stones in participants with diabetes. We demonstrated for the first time that physical activity exerts a beneficial effect on diabetes kidney stone participants at high BMI. This result may provide new insights into the impact of reducing BMI on kidney stones and provide new ways to prevent kidney stones.

Kidney stones are one of the most frequent and common diseases in urology, with complex causes and high recurrence rates. With the in-depth research on the etiology of kidney stones, the role of metabolic syndrome in the pathogenesis of kidney stones has also received increasing attention. Related studies have found that the occurrence of kidney stones is closely related to lifestyle-related diseases such as obesity, hypertension, dyslipidemia and hyperglycemia (22, 23). It has been reported in the literature that 48.7% of kidney stone patients have metabolic syndrome and the prevalence of kidney stones in patients with metabolic syndrome is 7.5 to 8.8% (24, 25).

Diabetes mellitus is a common metabolic disease, and some studies have found that there may be a common pathophysiological mechanism between the formation of kidney stones and the development of diabetes mellitus (26). The interconnection between diabetes mellitus and kidney stones is mainly due to the effect of insulin resistance on urinary pH and and the transport of ammonia and calcium in the kidney, which affects the production and transport of ammonium, causing a decrease in urinary pH (27, 28). The decrease in dissociation of uric acid in an acidic environment leads to increased precipitation and the formation of stones. Domingos et al. (13) found a higher prevalence of diabetes mellitus in patients with kidney stones compared to normal population (OR = 1.475, 95% CI, 1.283–1.696, *p* < 0.001) through questionnaire analysis of 23,349 individuals. The prevalence of kidney stones in diabetes patients was found to be 16.3% in this study, which is much higher than the prevalence of kidney stones in the normal population (9.3%) (4). This result is consistent with the findings of Taylor et al. (12) who found that diabetes patients are more likely to develop kidney stones than the general population.

Obesity and kidney stones obesity is a public health problem in many countries. Recent studies have shown that 34.8 to 41% of patients with kidney stones are also obese and that obesity is associated with an increased prevalence and recurrence of kidney stones (29, 30). Meanwhile, BMI is a common indicator used to define obesity. It has been found that BMI is positively correlated with calcium, oxalate, citrate, uric acid, sodium, potassium and phosphate in the urine, and the pH of the urine decreases with increasing BMI (31). Furthermore, even in patients with BMI <30 kg/m^2^, the higher the BMI, the greater the chance of kidney stones. Similar results were found in our study, where the risk of kidney stones was 1.514 (95% CI, 1.134–2.022, *p* = 0.005) for participants in the BMI ≥30.0 kg/m^2^ group compared to BMI <25.0 kg/m^2^ group.

Expect for metabolic factors, lifestyle such as smoking, alcohol consumption and physical activity have an important impact on the prevalence of kidney stones. However, there are relatively few studies on the relationship between physical activity and kidney stones, and the results are inconsistent (32). Three groups of studies reported a statistically significant protective effect of physical activity on kidney stones (19, 33, 34), and conversely, three studies reported no effect of physical activity on the risk of kidney stones (18, 29, 35). Sorensen et al. (29) found that physical activity reduced the risk of stones in women by 16%-31%. In addition, Zhuo et al. (35) found that duration of physical activity was an independent risk factor for kidney stones (OR = 0.840; 95% CI, 0.808–0.973) in a survey of 1,519 people in Southern China.

To our knowledge, the present study is the first to explore the association between physical activity, BMI, and diabetes kidney stones. Combined with interaction analysis, we found an interaction between physical activity and BMI on the occurrence of kidney stones. The results of the interaction between BMI and kidney stones indicated that the risk of kidney stones increased with increasing BMI but decreased with participation in physical activity. We also found that the proportion of <60 years, college or above, BMI <30 kg/m2, no hypertension, and never smoking was significantly higher in the physical activity group than in the no physical activity group. In addition, participants in the physical activity group had lower levels of blood urea nitrogen, creatinine and higher levels of eGFR compared to the no physical activity group. The above results suggest that physical activity can bring beneficial physiological aspects. In addition, some studies have found that physical activity can reduce the incidence of diabetes, hypertension, and obesity, which may explain why physical activity can reduce the incidence of kidney stones (36–38).

Although we have found significant modifications of the effect of physical activity on the effect of high BMI on participants with diabetes kidney stones, the study still has some limitations. First, this is a cross-sectional study and causality is difficult to verify. In addition, the NHANES database is a retrospective study with its inherent limitations. Finally, we did not provide the type of kidney stones and physical activity may have different outcomes for different stone types.

## Conclusion

Our study found that high BMI was a risk factor for participants with diabetes kidney stones and that physical activity moderated this relationship to some extent, with physical activity leading to beneficial physiological aspects. This result may provide new insights into the impact of reducing BMI on kidney stones and offer new approaches to kidney stone prevention.

## Data Availability Statement

The datasets presented in this study can be found in online repositories. The names of the repository/repositories and accession number(s) can be found in the article/supplementary material.

## Ethics Statement

This study was carried out in accordance with the recommendations of NHANES Committee with written informed consent from all subjects. All subjects gave written informed consent in accordance with the Declaration of Helsinki. The protocol was approved by the NHANES Committee. The patients/participants provided their written informed consent to participate in this study. Written informed consent was obtained from the individual(s) for the publication of any potentially identifiable images or data included in this article.

## Author Contributions

WM, XZ, GZ, and MC designed the research. WM, LZ, SS, and JW performed the research and analyzed results. WM, LZ, and SS wrote the paper. WM, LZ, XZ, GZ, and MC edited the manuscript and provided critical comments. All authors read and approved the final manuscript.

## Funding

This work was supported by National Natural Science Foundation of China (81900618 and 82170703), Tai-Shan Scholar Program from Shandong Province (tsqn202103116), Jiangsu Provincial Key Research and Development Program (BE2019751), Innovative Team of Jiangsu Provincial (2017ZXKJQW07), and the National Key Research and Development Program of China (SQ2017YFSF090096).

## Conflict of Interest

The authors declare that the research was conducted in the absence of any commercial or financial relationships that could be construed as a potential conflict of interest.

## Publisher's Note

All claims expressed in this article are solely those of the authors and do not necessarily represent those of their affiliated organizations, or those of the publisher, the editors and the reviewers. Any product that may be evaluated in this article, or claim that may be made by its manufacturer, is not guaranteed or endorsed by the publisher.
